# Escaping the Ashby limit for mechanical damping/stiffness trade-off using a constrained high internal friction interfacial layer

**DOI:** 10.1038/s41598-018-20670-0

**Published:** 2018-02-06

**Authors:** A. P. Unwin, P. J. Hine, I. M. Ward, M. Fujita, E. Tanaka, A. A. Gusev

**Affiliations:** 1 Soft Matter Physics Group, School of Physics and Astronomy, University of Leeds, Leeds, LS2 9JT UK; 2 The Kaiteki Institute, Mitsubishi Chemical Holdings, 1-1 Marunouchi 1-chome, Chiyoda-ku, Tokyo Japan; 3 Institute of Polymers, Department of Materials, ETH Zürich, 8093, Zürich, Switzerland

## Abstract

The development of new materials with reduced noise and vibration levels is an active area of research due to concerns in various aspects of environmental noise pollution and its effects on health. Excessive vibrations also reduce the service live of the structures and limit the fields of their utilization. In oscillations, the viscoelastic moduli of a material are complex and it is their loss part – the product of the stiffness part and loss tangent – that is commonly viewed as a figure of merit in noise and vibration damping applications. The stiffness modulus and loss tangent are usually mutually exclusive properties so it is a technological challenge to develop materials that simultaneously combine high stiffness and high loss. Here we achieve this rare balance of properties by filling a solid polymer matrix with rigid inorganic spheres coated by a sub-micron layer of a viscoelastic material with a high level of internal friction. We demonstrate that this combination can be experimentally realised and that the analytically predicted behaviour is closely reproduced, thereby escaping the often termed ‘Ashby’ limit for mechanical stiffness/damping trade-off and offering a new route for manufacturing advanced composite structures with markedly reduced noise and vibration levels.

## Introduction

In a recent study entitled ‘Noise pollution: A modern plague’ Goines and Hagler^1^ noted how noise pollution could have a significant impact on well-being and health and proposed a link between noise threshold and what they termed ‘displaced aggression’. Dzhambov and Dimitrova^2^ made similar links between noise, which they defined as unwanted sound, and a negative impact on health. They concentrated on what they called ‘street level’ noise. McAlexander *et al*.^3^ also investigated the effect of street level noise which they mostly attributed to vehicular traffic. Their study concluded that excessive noise could be potentially dangerous as an important risk factor for impacting negatively on health. They also identified traffic as one of the major sources of noise pollution and concluded that as it was difficult to escape noise exposure (in this case in parks and between buildings in New York City), new materials to effect noise levels could be very advantageous. Similar studies by Buxton *et al*.^4^, Pirrera *et al*.^5^ and Moudon^6^ have further confirmed this strong link between noise and health.

The reduction of noise can be realised in two main (and distinct) ways, either by absorbing the sound waves during their propagation or by damping the amplitude of the vibrating structure at the source. While there are a number of reported examples of materials that are able absorb sound waves, these normally involve specifically designed meta-materials, with often complicated internal structures, which are not easily applied to most structures. For this reason, in this work we have focussed our study on the second strategy, which is to rapidly decrease the amplitude of a vibrating structure. Additionally we have targeted an increase in the damping of a material, without sacrificing stiffness.

In general, materials with high mechanical damping rely on activating or making use of a mechanism of internal friction or hysteresis. This can include cracks in ceramics rubbing together to dissipate energy, reversible transformations in shape memory alloys^7^ (the transition from austenite to martensite) and destruction and construction of intercomponent hydrogen bonds^8^. However one of the most common methods is to utilise relative chain motions in polymers^9,10^. For polymers above their glass transition, increased mobility of both main chain (segmental) and side groups leads to viscoelasticity and high mechanical damping though also leading to a low storage modulus.

The use of materials for vibration damping applications has been described by Chung^11^ who reviews some of the key issues in this area of research. A key conclusion is that unwanted vibrations can be reduced by either increasing the damping (usually characterised by a measurement of the loss tangent tan*δ*) or by increasing the stiffness. The loss modulus is the product of these two quantities and was considered by Chung, as a possible figure of merit for the ability of a material to reduce vibrations. Another recent example is the work of Zhou *et al.*^12^ who studied the damping properties of polyacrylate emulsion/hindered phenol hybrids. This work describes the production of organic hybrids with a high mechanical loss, but accompanied, as is often the case where polymeric materials are used in the range of their glass transition, by a significant drop in the stiffness. Moore *et al.*^13^, in a purely theoretical study, modelled the effect of complex microstructure on the properties of filled elastomers, searching for possible structures that would have highly enhanced damping without sacrificing stiffness. Recently, a number of authors have reported metal/metal composite materials that combine increased damping with increased stiffness. Lakes *et al.*^14,15^ reported that the incorporation of inclusions that could change shape would produce materials with extreme values of modulus (higher than either constituent) and also high damping. Hussein and Frazier^16^ proposed periodic acoustic metamaterials (with local resonance properties) which would show high levels of damping without sacrificing stiffness.

So this combination of high damping and high stiffness is the key aim of our work reported here, implemented in a polymer based composite material. We exploit previously developed numerical micromechanics methods to find a composite system that demonstrates enhanced damping and stiffness and then manufacture this system and prove, experimentally, that these predicted properties can be realised. In a number of our previously published papers, Gusev utilised analytical micromechanics models and numerical finite element techniques^17–21^ to examine different material configurations for improving the energy dissipation of filled polymeric materials. While various systems have been studied numerically, including the use of rigid and viscoelastic spherical inclusions, the most promising combination as exemplified in^21^, consists of a solid polymer matrix filled with rigid inorganic spheres coated with a thin layer of a viscoelastic polymer (in the region of its glass transition temperature). In such a system, the thin viscoelastic coating is consequently sandwiched between the two rigid components, leading to a substantial amplification of the mechanical damping without seriously reducing the overall composite stiffness. Most importantly, the theoretical predictions suggested that there was a preferred (or optimum) thickness for the coating layer to balance these two competing effects.

## Methods

### Materials

In these experiments barium titanate beads (grade UB-02 M) from the Unikita Corporation were used. These were chosen as they have a narrow diameter distribution, and also a relatively large size (average diameter = 42 μm; bulk modulus = 142 GPa and shear modulus = 47 GPa were taken from^22^), which is an advantage when trying to achieve a small value of the ratio between the coating thickness, Δ, and the particle diameter, *R*. The target Δ/*R* was ~0.01 so an achievable coating thickness of ~200 nm for a 42 μm diameter particle. The viscoelastic coating used was Hydran AP40F made by the DIC Corporation. This was a water soluble polyurethane (and appropriate for spray coating) with a glass transition temperature above room temperature but below that of the polystyrene matrix. This material is normally employed as an adhesive, so it provides excellent compatibilisation between the barium titanate beads and the polystyrene matrix. The polystyrene grade was BASF PS2.

### Coating

The beads were coated with the polyurethane using a POWREX spray coater (MP MultiPlex). This machine uses a fluidized bed combined with a tangential spray in the lower section to give efficient particle coating. The target coating thickness was around 200 nm, which required a 0.22% suspension of the polyurethane. The coating fraction was subsequently measured by nitrogen analysis to be 0.17% on the particles. Spraying was carried out at 30 °C followed by drying at 80 °C.

### Composite manufacture

Blends of the coated beads and polystyrene were manufactured using a Europrism twin screw extruder. The extruder had a length to diameter screw ratio of 40:1 and used a constant screw speed of 300 rpm and a barrel temperature of 220 °C. The polystyrene was introduced at the front of the extruder, while the coated beads were introduced, using a powder feeder, two thirds of the way along the barrel. The speed of the powder feeder was varied to achieve each desired bead volume fraction (in this study 21%, 27%, 32% and 35%). The blended material was passed directly into a water bath and then chopped into pellets. The volume fractions were confirmed after manufacture using a gravimetric method.

### Preparation of samples for testing

Pellets of the blended composites were placed between brass plates in a hot press set at 180 °C and then pressed for four minutes before slow cooling to room temperature while still under pressure. Spacers were placed between the brass plates to achieve the desired thickness for the torsion tests. A pure PS sample was also manufactured using the same technique. A very important issue for the numerical modelling was to have experimental data on the properties of the pure coating material. As this is supplied as a water suspension, an amount of liquid was placed into a container and then left to dry naturally. This process was repeated until a thick enough sample (~1.4 mm) was produced, which could then be tested.

### Dynamic mechanical testing (DMTA)

The viscoelastic tests were carried out in rectangular torsion using a Rheometrics Dynamic Spectrometer RDS II. Samples of the required dimensions (10 mm wide, 1.4 mm thick and 55 mm long) were cut from the compression moulded sheets. Samples were tested over a range of temperatures (between +30 and +110 °C) at a frequency of 1 Hz and an oscillatory strain of 0.05%. This level of strain was chosen so as to achieve a balance between producing a high enough strain to give a measurable force, while remaining in the linear regime and keeping the normal force to a minimum.

### Scanning electron microscope (SEM) investigation

Details of the particles and the coated particles, and the morphology of the multi-phase composites, were studied using a Hitachi SU8230 scanning electron microscope. The as received and coated particles were directly imaged while the coating thickness was viewed by embedding the three phase composite into epoxy resin and polishing, finishing off with a 0.5 µm Al_2_O_3_ suspension. Finally a freeze fractured surface was prepared by immersing in liquid nitrogen before fracturing. All samples were coated with a few nanometres of platinum before imaging.

## Results

### Numerical micromechanics simulations for the present study

**Figure 1:**
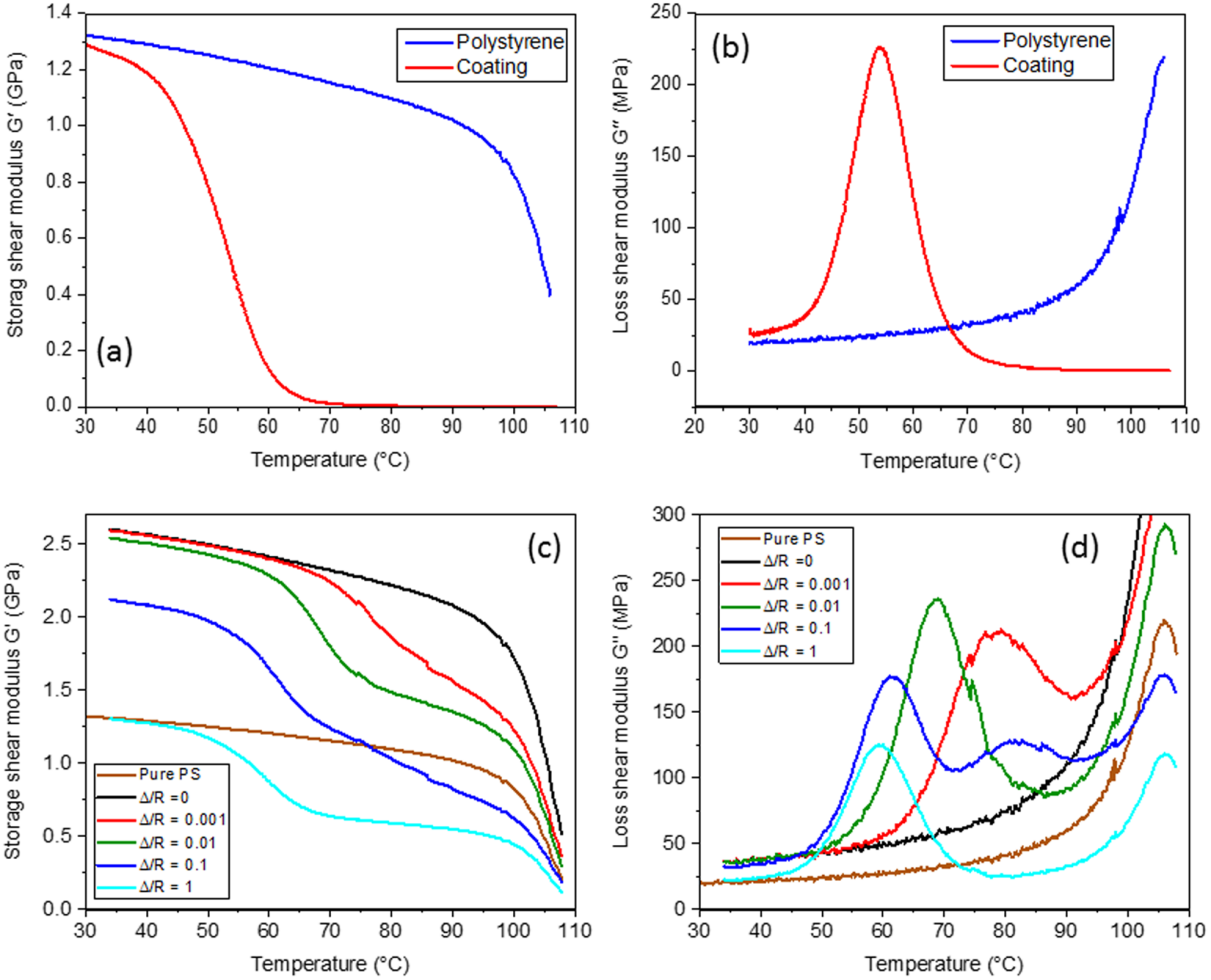
Motivation for the current experimental study. (**a**) and (**b**) The storage and loss moduli for the composite constituents, namely the viscoelastic polyurethane coating and the solid polystyrene matrix. (**c**) Analytical predictions for the storage shear modulus *G*′ of a random dispersion of coated glass spheres of radius *R* with different coating thickness, Δ, at a volume fraction of spheres of *c* = 0.3. (**d**) A similar prediction but for the loss shear modulus *G*′′.

The starting focus for the current work was provided by numerical simulations based on the optimum microstructure previously established by Gusev^21^, of rigid spheres coated with a sub-micron viscoelastic layer embedded in a polymer matrix. The only difference is that here we used the experimentally measured phase properties for the various components (the previous study used ‘model’ material properties). A water soluble polyurethane (Hydran AP40F) was chosen as the viscoelastic coating while the base matrix was a standard polystyrene (BASF grade PS2). The storage and loss shear modulus of these two materials (*G*′ and *G*′′, respectively) was measured in torsion over a temperature range of 30 °C to 110 °C and at a frequency of 1 Hz (for experimental details see^23^ and the Methods section) and these results are shown in Fig. 1a and b. It can be seen that the coating has a glass transition temperature (a viscoelastic loss peak) at ~50 °C but below that of the polystyrene.

In terms of determining the optimum design, there are a number of important variables to simultaneously study, including the phase properties, the particle volume fraction, *c*, and the viscoelastic layer thickness, Δ. While finite element techniques are the ultimate method for prediction, a validated analytical solution can offer a much more time effective design process. In three recent papers^19–21^, Gusev reported that the predictions of viscoelastic behaviour could be equally well achieved using simple analytical micromechanics schemes. First, it was illustrated that the generalized self-consistent (GSC) model of Christensen and Lo gave almost identical predictions of elastic behaviour to finite element estimates, for rigid sphere filled polymers. Second, by linking together the *n-*layered model of Herve and Zaoui^24^ and the viscoelastic correspondence principle^13,25^, viscoelastic predictions of three phase systems could be equally well formed. This pairing was found to give almost identical predictions to finite element calculations, at a vastly decreased solution time, and so this combination has been employed to motivate the experimental work in this investigation. It was also shown, as discussed in these papers^19–21^, that an assumption of a non-lossy temperature independent bulk modulus of 4 GPa is rational and accurate for both the polymeric matrix and the coating, and as so this assumption has also been used in this current work.

Analytical predictions for the storage shear modulus *G*′ and the loss shear modulus *G*′′ for coated barium titanate spheres in polystyrene can be seen in Fig. 1c and d. Here the important parameter varied was the ratio of the coating layer thickness Δ to the particle radius *R* (keeping the overall sphere volume fraction fixed at a value of 30%). These two figures show that the maximum amplified damping occurs in the range of Δ/*R* of ~10^−2^. The suggestion is that if the coating is thinner than this ‘optimum’, then the volume of the viscoelastic material is lower and so the overall enhancement of the damping is reduced. Alternatively, if the coating is thicker than the ‘optimum’, then the strain concentration in the viscoelastic layer is less and hence the amplification of the damping is again lower. At a ratio of ~10^−2^, the amplification is at a maximum, coupled with a storage shear modulus significantly greater than the base polystyrene matrix. This combination of stiffness and damping is the key aim of this study. Finally, it is interesting to note that in the composites the maximum viscoelastic loss is predicted to occur at a higher temperature than in the bulk coating material (Fig. 1b).

Having carried out this first design optimisation step using the validated analytical model, the next stage was to manufacture samples in this optimum range of coating thickness, and then measure their viscoelastic properties and see if the proposed effects in Fig. 1c and d can be realised.

### Experimental Results

**Figure 2:**
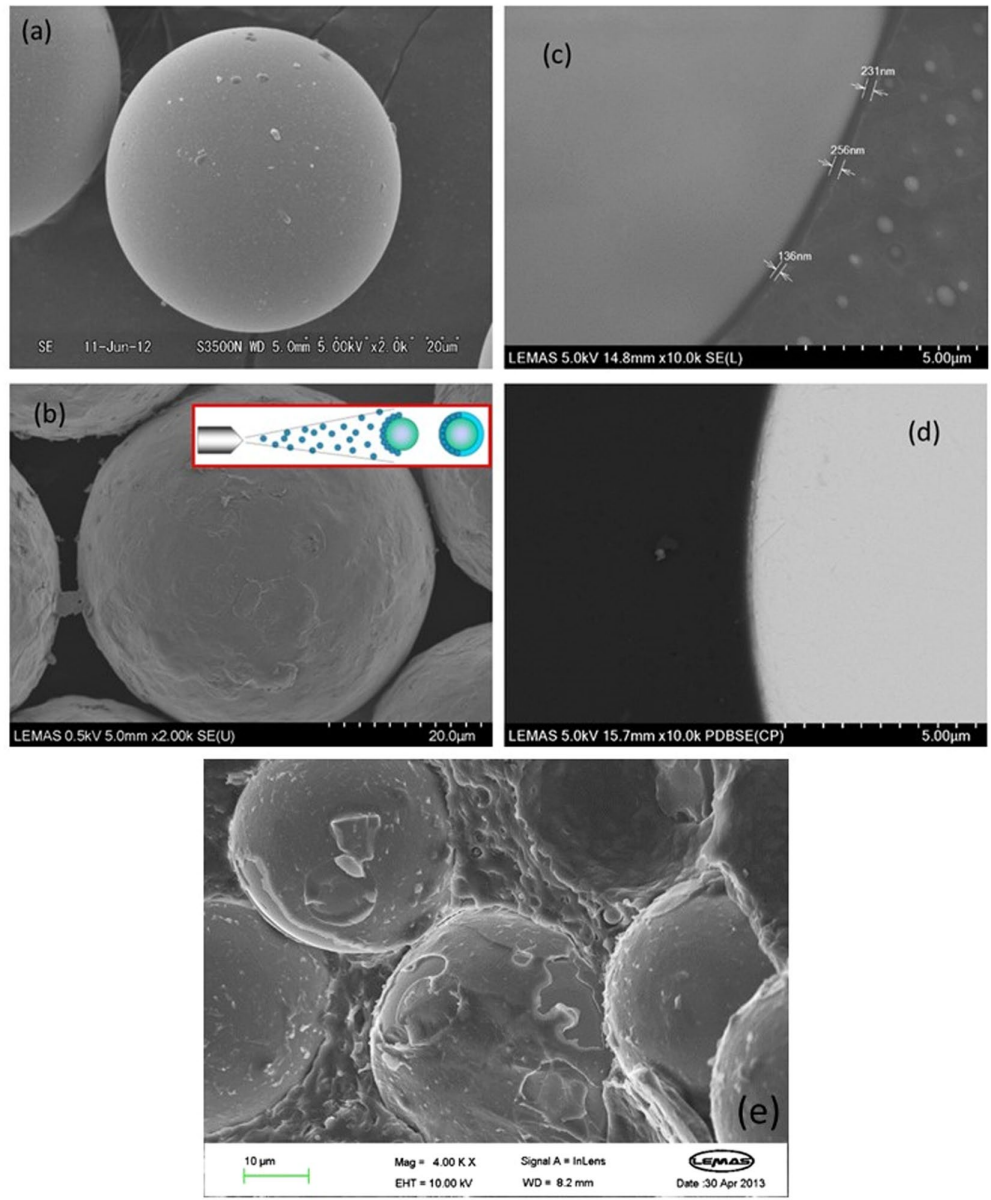
Scanning electron microscope images of the barium titanate beads and details of the viscoelastic coating layer. (**a**) The as received uncoated beads, average diameter = 42 μm. (**b**) A typical bead after spray coating using a water emulsion of the coating. (**c**) Higher magnification picture of a coated bead embedded in PS suggesting a layer thickness of 200 nm. (**d**) An alternative contrasting technique confirming the layer thickness. (**e**) A freeze fracture surface from the final composite again confirming the presence of the layer after processing.

As described above, the coated particle system comprised three components. For the coating, a number of spraying experiments (for more details see the Methods) were carried out using the chosen water soluble polyurethane, and it was established that a dried coating thickness of the order of a few hundred nanometres could be readily achieved. The second component was the base matrix material. Here we chose a standard amorphous polystyrene whose dynamic properties were measured to be constant through the range of the glass transition of the coating (Fig. 1b). The third component was the stiff filler. To achieve the suggested optimum ratio of the coating thickness/particle ratios of ~10^−2^ from Fig. 1c we specifically chose a particular grade of barium titanate beads that had an average diameter of 42 μm. In this way by choosing a larger particle than would normally be employed, we were able to achieve close to the predicted optimum ratio of coating thickness to particle radius. Fig. 2 displays a number of micrographs of the beads using scanning electron microscopy. Figure 2a has a picture of the as received uncoated beads, while Fig. 2b is a picture of a typical spray coated bead. Figure 2c and d are SEM micrographs from polished sections taken from the final composite, indicating that the thickness of coating that survives the high shear and high temperature processing is of the order of 200 nm. Finally Fig. 2e shows a freeze fractured sample, again revealing evidence of the coating layer and its typical thickness of a few hundred nanometres.

**Figure 3:**
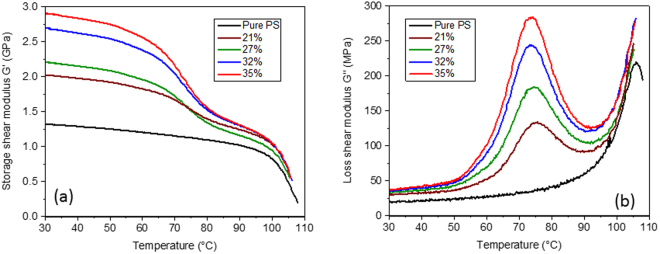
Experimentally measured dynamic temperature scans. (**a**) Storage shear modulus for coated particle filled samples at particle volume fractions from 21% to 35%. (**b**) Loss shear modulus for coated particle filled samples at particle volume fractions from 21% to 35%.

Composites were manufactured from these coated beads (as described in the Methods section) at particle volume fractions ranging from 21% to 35%. Figure 3a and b show experimental dynamic temperature scans (for details of the tests see the Methods section) carried out in torsion for these range of samples. Measurements were carried out over a temperature range designed to pass through the glass transition region of the viscoelastic coating (30 °C to 110 °C), the same range as for the individual phases, see Fig. 1a and b. As the volume fraction of the coated beads is increased, it is seen that both the storage modulus *G*′ and the loss modulus *G*′′ increase monotonically. For the highest particle fraction of 35%, the peak loss modulus was measured to be over an order of magnitude greater than the pure PS sample, while the storage modulus was close to double that of pure PS, offering the possibility of a material with a rare combination of high stiffness and high damping.

Next, of course, it was of great interest to compare how the experimentally measured viscoelastic properties in Fig. 3a and b compared to numerical predictions based on the actual phase properties. A key issue is, what is the most appropriate value for Δ/*R*? The model predictions for *G*′′ (Fig. 1d), indicated how sensitive the peak position is to this ratio, rising from ~50 °C for the bulk sample (Fig. 1b) to ~80 °C for a value of Δ/*R* of 10^−3^. For this reason, we first carried out numerical simulations for only one particle volume fraction (35%), but for three values of Δ/*R* equal to 0.001, 0.005 and 0.01.

**Figure 4:**
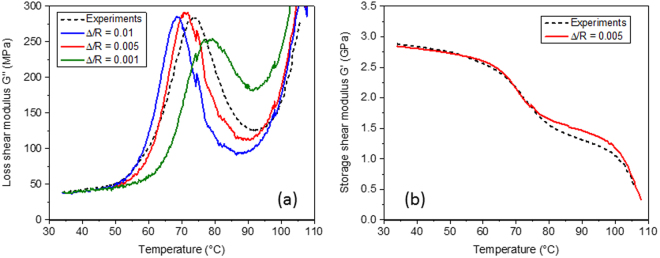
Numerical predictions for the three component system – bead fraction *c* = 0.35. (**a**) Numerical temperature scans for loss shear modulus *G*″ at constant Δ/*R* values (0.01, 0.005 and 0.001) compared to the experimental measurements, which is the dotted black line. (**b**) Temperature scans for storage shear modulus *G*′ at Δ/*R* = 0.005.

Figure 4a shows the numerical results for the loss shear modulus *G*″ along with the experimental measurements which are indicated by a dotted black line. It is seen that the agreement between the experimental measurements and the numerical predictions is generally excellent and that the best agreement (both for the peak position and peak amplitude) is for a value of Δ/*R* of around 0.005. Figure 4b then shows the comparison for the storage shear modulus *G*′ but for only one value of Δ/*R* = 0.005. The agreement is also seen to be excellent for this parameter. As discussed earlier, if the thickness of the coating is too low (lower than the ratio of Δ/*R* = 0.005), then the peak amplitude decreases and the peak position moves to a higher temperature, while if the coating is thicker, then the peak position moves to a slightly lower temperature. Given the simplicity of the Herve and Zaoui^24^ generalization of the Christensen and Lo model^26^, and the fact that the modelling only considers micromechanical effects, the agreement between the measured properties and predictions (both for the loss peak position, peak amplitude and peak width) has to be considered to be striking. We would not expect the agreement to be perfect, given that the coating thickness is both non-uniform and approaching nanoscale dimensions. It is clear that the ‘effective’ coating thickness required by the model to predict the experimentally measured viscoelastic properties is in close agreement with that seen in the actual materials (of the order of a few hundred nanometres) as a value of Δ/*R* = 0.005 would suggest an effective coating thickness of ~100 nm, cf. Fig. 2c. It is clear that the model could be further used to explore other potential material combinations to further enhance the mechanical damping effects by using various optimum microstructural design strategies as reported in^21^.

**Figure 5:**
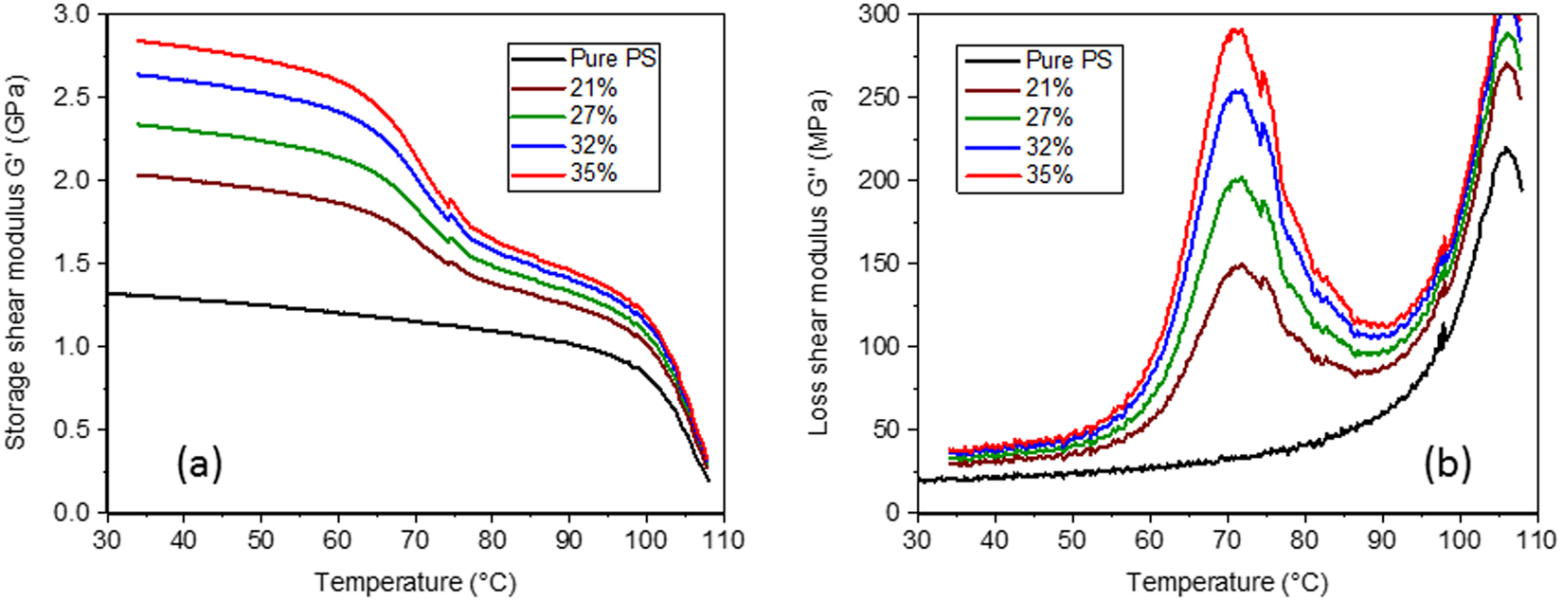
Numerically predictions based on the phase properties from Fig. 1a and b. (**a**) Predicted storage shear modulus for coated particle filled samples at particle volume fractions from 21% to 35% using a value of Δ/*R* = 0.005. (**b**) Loss shear modulus for coated particle filled samples for the same particle volume fractions and value of Δ/*R*.

Finally, in Fig. 5a and b we show numerical predictions for all the bead fractions (from 21% to 35%) based on the measured phase properties from Fig. 1a and b and using a value for Δ/*R* of 0.005. The agreement between the experimental results (Fig. 3a and b) and the numerical predictions on Fig. 5a and b can be seen to be striking, both in terms of the amplitude and the position of the loss peak, at all particle volume fractions.

## Discussion

One key aim of this study was to develop materials that would be able to quickly damp the amplitude of any induced structural vibrations. In a recent paper^21^, we have studied damped vibrations of viscoelastic Bernoulli-Euler beams and Kirchhoff plates. This paper reported that neglecting the Poisson’s ratio effect, the material’s contribution to the natural vibration frequencies of the viscoelastic beams and plates is of the same form, i.e.,

1 $${\omega }_{p}^{\ast }={\omega }_{p}^{\prime} +i{\omega }_{p}^{^{\prime\prime} }\propto \sqrt{{E}^{\ast }/\rho }$$

where *p* is an integer labelling the natural modes, E^*^ = *E*′ + *iE*″ is the complex viscoelastic Young’s modulus and *ρ* is the density. The proportionality coefficient is real and it is determined by the geometry of the studied beam (or plate), imposed boundary conditions and the mode number *p*. The corresponding mode shapes are also all real and they are simply those of the comparable elasticity problem^21^. In free vibrations, the normal mode dynamics is of a damped exponential form, with the mode frequencies ${\omega }_{p}^{\prime}$ and the mode amplitudes evolving as

2 $${A}_{p}(t)={A}_{p}(0)\exp (-t/{\tau }_{p})$$

where *A*_*p*_(*t*) is the amplitude of mode *p* at time *t* and *τ*_*p*_ = 1/*ω*″_*p*_ the mode damping time. The vibration amplitude decay is then directly related to the emitted sound pressure levels whereas the sound intensity (loudness perception) is proportional to the amplitude squared.

**Figure 6:**
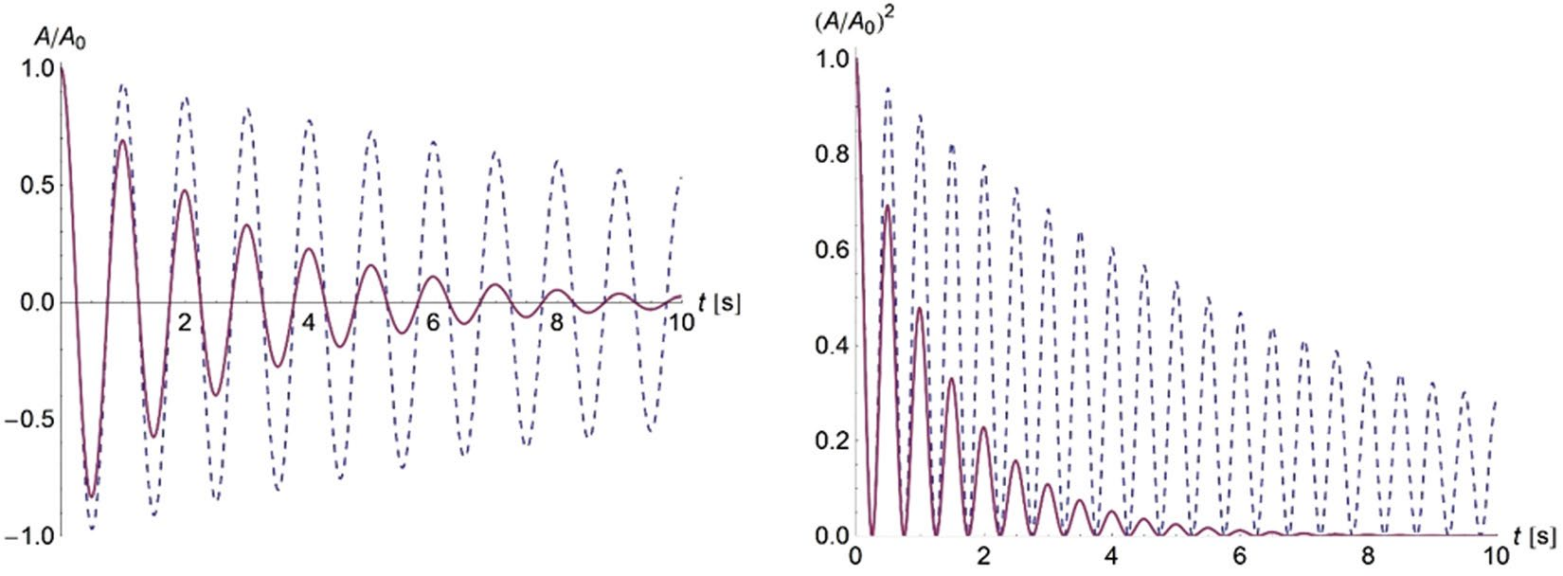
Amplitude decay of the fundamental mode of vibration for simply supported square plates made up of viscoelastic composites with uncoated (dashed lines) and coated (solid lines) beads. The plates have a fundamental frequency of 1 Hz, which is achieved by adjusting their edges *L* to 1.32 m (coated beads) and 1.25 m (uncoated beads). Shorthand notation *A* = *A*_0_(*t*) and *A*_0_ = *A*_0_(0) is used. The required viscoelastic plane stress-reduced stiffness coefficient Q^*^ is calculated from numerically predicted bulk and shear moduli, K^*^ and G^*^, respectively^21^, using a standard elasticity relation Q^*^ = 4G^*^(3K^*^ + G^*^)/(3K^*^ + 4G^*^).

To illustrate the vibration damping performance achievable with the manufactured composite materials developed here, we consider two simply supported square plates of edge length *L* and thickness *h* = 1 mm made up of the polystyrene matrix reinforced by either uncoated or coated barium titanate beads dispersed at a volume fraction of 35%. Assuming a coating thickness of Δ/*R* = 0.005 as discussed in Figs 4 and 5, we use numerically predicted effective viscoelastic moduli at the *G*″ peak at 72 °C to estimate the fundamental mode damping times of the plates while tuning their fundamental frequencies to 1 Hz (*ω*′_0_ = 2*π*) by adjusting their edge lengths *L*^21^. Figure 6 shows that using composites with coated beads, substantial improvements can be achieved in damping structural vibrations and hence also in cutting their emitted sound noise levels.

**Figure 7:**
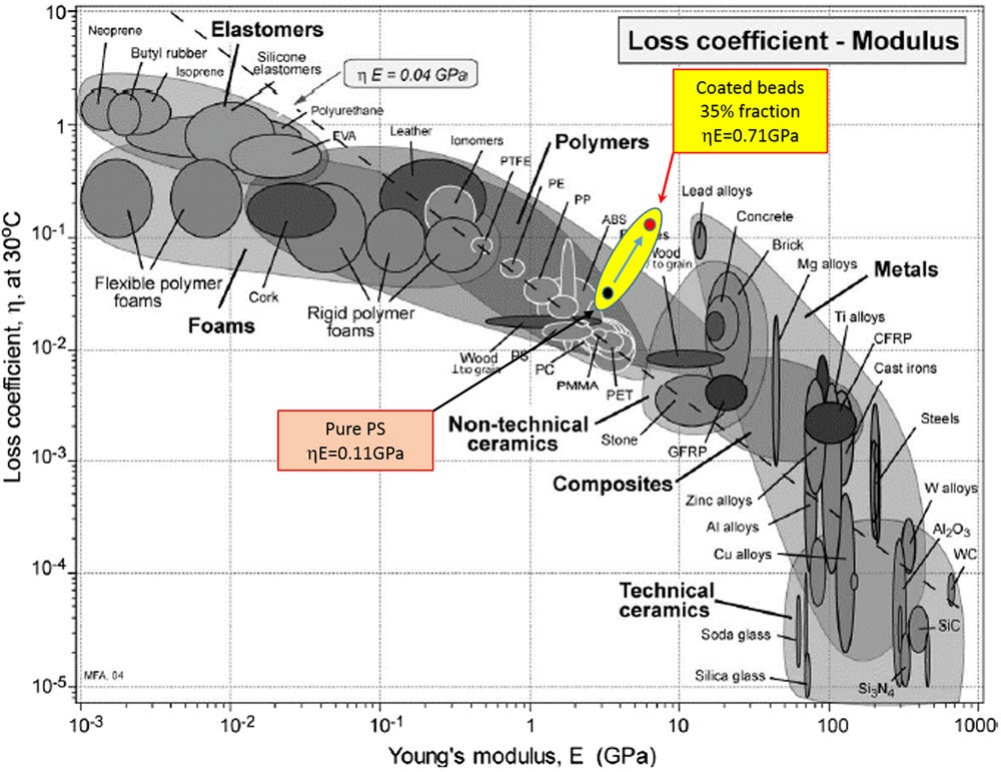
The Ashby log-log plot for loss coefficient *η* = *E*″/*E*′ versus Young’s modulus *E*′. Superimposed on this plot are the extreme results from the current study. The black circle is the result for pure polystyrene, while the red circle is the result for polystyrene reinforced with 35% coated glass beads.

The other goal of this study was to achieve increased damping without sacrificing stiffness, and to design materials that combined both high damping and high stiffness. A very useful way to visualise the normal trade-off between two different properties is from the work of Ashby^27,28^, who collated data from a vast range of materials. From this data, Ashby created a range of log/log plots for different important pairs of properties in order to produce a range of Materials Selection Charts for designers to use. For selecting materials for either low or high damping, Ashby created a log/log plot of loss coefficient *η *(=tan *δ*) against Young’s modulus (at a temperature of 30 °C), and this is represented in Fig. 7. This plot indicates that a compromise is usually required between these two properties, as materials with high damping have a low modulus and vice-versa. It is seen that the results roughly cluster around a dashed line with a value of the loss modulus *E*″ = 0.04 GPa. So for a typical polymer with a storage Young’s modulus *E*′ of 3 GPa, this selection chart suggests an extreme range for the loss modulus *E*″ between 0.03 and 0.3 GPa.

To place the materials developed here in this context, requires a value for both storage Young’s modulus *E*′ and its tan *δ* at 30 °C. However in this work, only the storage shear modulus *G*′ and its tan *δ* were obtained directly from the mechanical measurements and the peak performance is at 76 °C, not at 30 °C. We have used two different methods to assess appropriate values for plotting on Fig. 7. The first method uses the common rule of thumb often employed for a quick practical conversion, namely that the viscoelastic Young’s and shear moduli of polymers have the same tan*δ* whereas their storage parts are related by *E*′ ≈ 3*G*′. Table 1 shows the values of the calculated Young’s modulus, *E*′, and tan *δ* using this approximation. These could be considered appropriate for plotting on Fig. 7, and despite the fact that the temperature is different, the values would be expected to represent results for a coating with a lower glass transition temperature, and hence peak properties at 30 °C.

**Table 1:** Measured and calculated peak viscoelastic moduli for composites with the various particle fractions manufactured in this study, assuming *E*′ ≈ 3*G*′. The measured moduli are given at a chosen temperature of 76 °C, which is the peak position for most of the composites’ samples.

Particle fraction [vol. %]	*G*′ and tan*δ* at 76 °C		*E*′ and tan*δ* at 76 °C	
	*G*′ [Gpa]	tan *δ*	*E*′ [Gpa]	tan *δ*
0	1.12	0.033	3.36	0.033
21	1.49	0.088	4.47	0.088
27	1.43	0.122	4.29	0.122
32	1.76	0.137	5.28	0.137
35	1.78	0.153	5.34	0.153

A second method to obtain appropriate values for plotting on Fig. 7, is to use the previously validated numerical model. The calculated values of the storage shear modulus *G*′ and loss shear modulus *G*″ are seen in Fig. 5 to be very similar to the experimental measurements shown in Fig. 3, although the peak in *G*″ (and hence tan*δ*) is at a slightly lower temperature of around 72 °C. We suggest that the viscoelastic moduli of solid amorphous polymers are similar when represented in terms of temperature deviation from their glass transition temperatures,*T*_*g*_, and so we have shifted the measured coating temperature scan of Fig. 1b by 42 °C so that the assumed *T*_*g*_ is now 8 °C, and using the unshifted former measured scan for PS matrix, numerically predicted the required peak values, which now all occurred at 30 °C. The results (which provide a direct calculation of the storage Young’s modulus *E*′ and tan *δ*) are given in Table 2 and it is seen that they are very similar to those in Table 1 (particularly when considered on a log-log evaluation as Fig. 7). As this second method is slightly more rigorous, we decided to present the results from Table 2 on Fig. 7. The extreme values for the samples studied here, which is pure polystyrene (black circle) and polystyrene with 35% coated spheres (red circle), are now shown plotted on the Ashby plot in Fig. 7.

**Table 2:** Calculated peak viscoelastic moduli for composites with the various particle fractions manufactured in this study. First at 72 °C, using the directly measured coating viscoelastic data and second, by shifting the coating data to give a peak in tan *δ* at 30 °C.

Particle fraction [vol. %]	*G*′ and tan *δ* at 72 °C		*E*′ and tan *δ* at 30 °C	
	*G*′ [GPa]	tan *δ*	*E*′ [GPa]	tan *δ*
0	1.14	0.030	3.57	0.013
21	1.57	0.095	4.87	0.072
27	1.73	0.116	5.30	0.091
32	1.88	0.134	5.75	0.107
35	1.98	0.148	6.04	0.117

It is seen that the results from the new materials produced here form a set that has a distinctively positive gradient on this log/log plot as the particle volume fraction (and hence also the fraction of the viscoelastic coating) is increased, in stark contrast to all the other material types. For the highest coated particle fraction achieved in this study of 35% (shown by the red circle), it is seen that this lies significantly outside the extreme range indicated by Ashby, thereby escaping the so called Ashby limit.

Figure 7 also shows that the upper limit of the damping for the new materials is at a similar level to lead alloys. However if the density of the various materials shown in Fig. 7 were taken into account, and therefore plotted as the ‘specific modulus’ on the *Y*-axis as discussed in ref.^21^, where a refined figure of merit for structural vibration damping applications was suggested based on the analysis of damped normal mode vibrations of viscoelastic beams and plates as given in Eq. (1) above, then the new structured composites would have a significant advantage, as they have a density of ~1500 kg/m^3^ compared to a value for lead of 11300 kg/m^3^.

Due to the a significant societal drive to reduce the weight of vehicles such as cars and aircraft, to improve energy efficiency and reduce unwanted noise and vibrations, we would expect there to be a number of possible applications for the new materials developed in this work^29^, based on their uncommon combination of high stiffness and high damping. The materials described here have the industrial potential to both save weight and significantly reduce unwanted vibrations. Other possible applications include the important reduction of unwanted sound and vibration generation within the living environment, such as office buildings or the home and also in components of precise electronic and audio equipment.

## Conclusions

In this study we have described the development of a new composite material that simultaneously exhibits the rare combination of high stiffness and high viscoelastic loss. Numerical simulations were first carried out (based on a model validated in a previous publication) to ascertain the optimum set of parameters that were predicted to give this behaviour. Then we proved that samples could be manufactured to this specification (comprising a polymer reinforced by glass spheres with a thin, sub-micron viscoelastic coating), and that their experimentally measured viscoelastic properties closely matched the analytical predictions. We demonstrated that such a material would be predicted to significantly reduce the amplitude decay time for the damped free vibration of a viscoelastic plate, compared to a similar plate made from a polymer reinforced with uncoated beads. Plotting the properties of the new materials on an Ashby plot of loss factor vs Young’s modulus, showed them to fall well above the trend line of all materials and thereby escaping the Ashby limit.
